# Hyponatremia: incidence, risk factors, and consequences in the elderly in a home-based primary care program 

**DOI:** 10.5414/CN108453

**Published:** 2015-06-03

**Authors:** Anirban Ganguli, Ryan C. Mascarenhas, Namirah Jamshed, Eshetu Tefera, Judith H. Veis

**Affiliations:** 1 Hospital of the University of Pennsylvania, Philadelphia, PA,; 2 Ochsner Medical Center, New Orleans, LA, and; 3 Medstar Washington Hospital Center, Washington, DC, USA

**Keywords:** hyponatremia, risk factors, geriatric outcomes

## Abstract

Aims: To determine the incidence, risk factors, etiology, and associations of hyponatremia in community-dwelling elderly with geriatric morbidity and mortality. Materials: Elderly participants of a single center home-based primary care program were included. Method: Retrospective chart review was conducted on demographic and clinical variables, comorbid diseases, frailty by Fried criteria and biochemical tests over a 1-year period. Primary outcome measure was a composite of falls, fractures due to falls, and hospitalization witnessed within the first year of enrollment into the program. Secondary outcome was all-cause mortality. Results: The study population (n = 608) had a mean age of 84.3 ± 9.3 years and was largely female (77.1%) and African-American (89.5%). Mean follow-up was 41.5 months. Frailty was seen in 44.4%. Incidence of all-cause mortality was 26.9%. Initial hyponatremia occurred in 8.71% (n = 53), and persistent hyponatremia (> 6 months of low serum sodium) in 4.1% (n = 25) of the study population. The major causes of hyponatremia included multiple potential causes, idiopathic syndrome of inappropriate anti-diuretic hormone (SIADH) and medications (thiazides and selective serotonin reuptake inhibitor (SSRI)). Primary outcome was independently associated with frailty (Odds ratio (OR) of 2.33) and persistent but not initial hyponatremia (OR 3.52). Secondary outcome was independently associated with age > 75 years (OR 2.88) and Afro-American race (OR 2.09) only but not to frailty or hyponatremia. Conclusions: Hyponatremia is common in home-bound elderly patients and its persistence independently contributes to falls, fractures, and hospitalization but not mortality. Our study highlights a new association of hyponatremia with frailty and underscores the need to study time-dependent association of hyponatremia with epidemiological outcomes.

## Introduction 

Hyponatremia is the most common electrolyte disorder in clinical practice [1], with higher incidence in the acute-care hospital, intensive care unit, or ambulatory-care setting as compared to a community-dwelling population [2]. Cross-sectional population studies show that the incidence increases with age [2, 3], with a reported point prevalence of 7.18% in the elderly versus 2.98% in a younger cohort [3]. Most studies [2, 4] suggest that hyponatremia encountered in the geriatric ambulatory care setting is milder compared to an inpatient population. Mild chronic hyponatremia, which is by far the most common form encountered in the outpatient population [1], has been recently implicated in cognitive and gait disturbances, osteoporosis, nonvertebral fractures, and falls [5, 6, 7, 8]. Furthermore, hyponatremia has been shown to contribute to all-cause mortality in the ambulatory geriatric population [9] besides being a known poor prognosticator in congestive heart failure (CHF) [10], cirrhosis [11], pneumonia [12], and in hospitalized patients [13, 14]. 

To our knowledge, the incidence and risk factors for hyponatremia in the frail elderly population has not been studied. The implication of such research is profound as this population is unique not only because of the burden of comorbid illness but also the high prevalence of disability, limited functional Status and frailty independently contributing to falls, injuries, disability, institutionalization, mood disorders, and other adverse health outcomes [15]. Given the importance and potential to treat hyponatremia, we decided to investigate the epidemiology of hyponatremia in a home-bound elderly patient population and study its impact on falls, fractures due to falls, hospitalization, and mortality. 

## Methods 

A retrospective chart review was conducted using the electronic medical records (EMR) of patients enrolled in the house call program (HBPC) at Medstar Washington Hospital Center (WHC), a community hospital and referral center in Washington, DC from 1^st^ January 2000 to 31^st^ December 2012. The HBPC program comprises an interdisciplinary team including physicians, nurse practitioners, social workers, and office staff. Patients are seen as medically necessary at home, and urgent visits are made within 24 hours. The program sees patients within a 5 mile radius of the sponsor hospital, WHC. Follow up visits vary from once a month to once every 4 months, depending on the acuity of care. The present study was approved by the institutional ethics committee and was in accordance with the declaration of Helsinki. All the provisions of Health Insurance Portability and Accountability Act (HIPAA) act were satisfied to meet patient confidentiality. 

We reviewed the records from the time of the first physician note in the EMR until 1 year after and included demographics, past medical problems, medications, hospitalizations, incidence of fall, fractures, or injuries related to fall, and terminal events. Race/ethnicity was as per documented in the EMR and was recorded in view of prior studies showing implications on the etiology of hyponatremia [7, 9]. Mortality data was collected until January 31^st^ 2012. Primary outcome was defined as a composite of falls, fractures (vertebral or nonvertebral, related or unrelated to a preceding history of falls), and hospitalization (related or unrelated to hyponatremia) recorded during the first year of enrollment into the house call program. Secondary outcome was all-cause mortality. We hypothesized that baseline or persistent hyponatremia was a predictor of both primary and secondary outcomes. Inclusion criteria included patients with age of more than 65 years enrolled in the house call program with complete medical records for at least 1 year. Exclusion criteria included patients whose follow-up data was less than 1 year. Two independent reviewers analyzed charts, and data was cross-checked to maintain internal consistency of results. 

Hyponatremia was defined as serum sodium < 136 mEq/L on 2 or more occasions (corrected for hyperglycemia using a correction factor of 1.6 mEq/L decrease in serum sodium concentration for every 100 mg/dL increase in plasma glucose concentration). Initial hyponatremia was defined on the basis of the first recorded Na after enrollment into the program, but not persisting for more than 6 months after the initial measurement. Persistent hyponatremia was defined as an Na < 136 mEq/L for more than 6 consecutive months during the study year. Mild hyponatremia was defined as serum sodium between 130 and 135 mEq/L, moderate hyponatremia as serum sodium between 125 and 129 mEq/L, and severe hyponatremia as serum sodium less than 125 mEq/L. Functional limitation was assessed by frailty using the Fried Index, which was present if the patient had at least 3 of the following [16]: documented unintentional weight loss (10 lbs or more in a year), self-reported exhaustion, weakness (grip strength), slow walking speed, and low physical activities. Causes of hyponatremia were assessed in detail, and additional charts were analyzed, this included renal consults for unexplained hyponatremia. Syndrome of inappropriate anti-diuretic hormone (SIADH) was diagnosed if the patient had hyponatremia with serum osmolarity less than 275 mosm/L, urine osmolarity > 100 mOsm/kg, urine sodium > 20 mmol/L, absence of significant renal dysfunction blood urea nitrogen < 10 mg/dL, and serum creatinine < 1.5 mg/dL clinical normovolemia and no other potential causes of normovolemic hyponatremia including thyroid and adrenal dysfunction. Comorbidity was assessed using the Charlson Comorbidity Index, which has been previously shown to predict 10-year mortality in a wide range of comorbid conditions (a total of 22 conditions) [17]. Each condition is assigned a score of 1, 2, 3, or 6, depending on the risk of dying associated with each one. Scores are summed to provide a total score to predict mortality. Clinical conditions, and associated scores are as follows:

- 1 each: Myocardial infarct, congestive heart failure, peripheral vascular disease, dementia, cerebrovascular disease, chronic lung disease, connective tissue disease, ulcer, chronic liver disease, diabetes.
- 2 each: Hemiplegia, moderate or severe kidney disease, diabetes with end organ damage, tumor, leukemia, lymphoma.
- 3 each: Moderate or severe liver disease.
- 6 each: Malignant tumor, metastasis, AIDS.

Chronic kidney disease was defined as persistent estimated glomerular filtration rate (GFR) of < 60 mL/min over a 3-month period as measured by the modification of diet in renal disease (MDRD) equation. 

### Statistical methods 

Continuous clinical variables were analyzed using the nonparametric Wilcoxon rank sum tests since normality assumption was not satisfied. χ^2^-tests were used to investigate differences in categorical clinical variables between hyponatremic and normonatremic groups. Logistic regression was used to determine independent predictors for primary and secondary outcomes. p-value of < 0.05 was considered significant. Statistical Analysis System software version 9.2 (SAS Institute Inc., Cary, NC, USA) was used to perform the analysis. 

## Results 

Out of a total of 958 patient charts, 608 patients were found eligible for analysis. Demographic variables are shown in Table 1. The mean age of the study population was 84.3 years, with the majority (77.1%) being female and African-American (89.5%). Mean follow up was 41.5 months. Notable comorbidities encountered included hypertension (86.7%), diabetes mellitus (35.4%), dementia (44.1%), psychiatric disease (32.5%), chronic kidney disease (26.6%), cerebrovascular disease (32.2%), congestive heart failure (26.3%), hypothyroidism (13.8%), and frailty by Fried Index (44.4%). Amongst patients with dementia (n = 266), the majority had Alzheimer disease (n = 159), followed by vascular dementia (n = 91), fronto-temporal dementia (n = 5), and unknown in 11 patients. A total of 94 patients (15.6%) were considered to have a terminal illness, which included malignancy in 30, advanced dementia in 40, and end-stage heart failure in 24 patients. 

**Figure 1.**
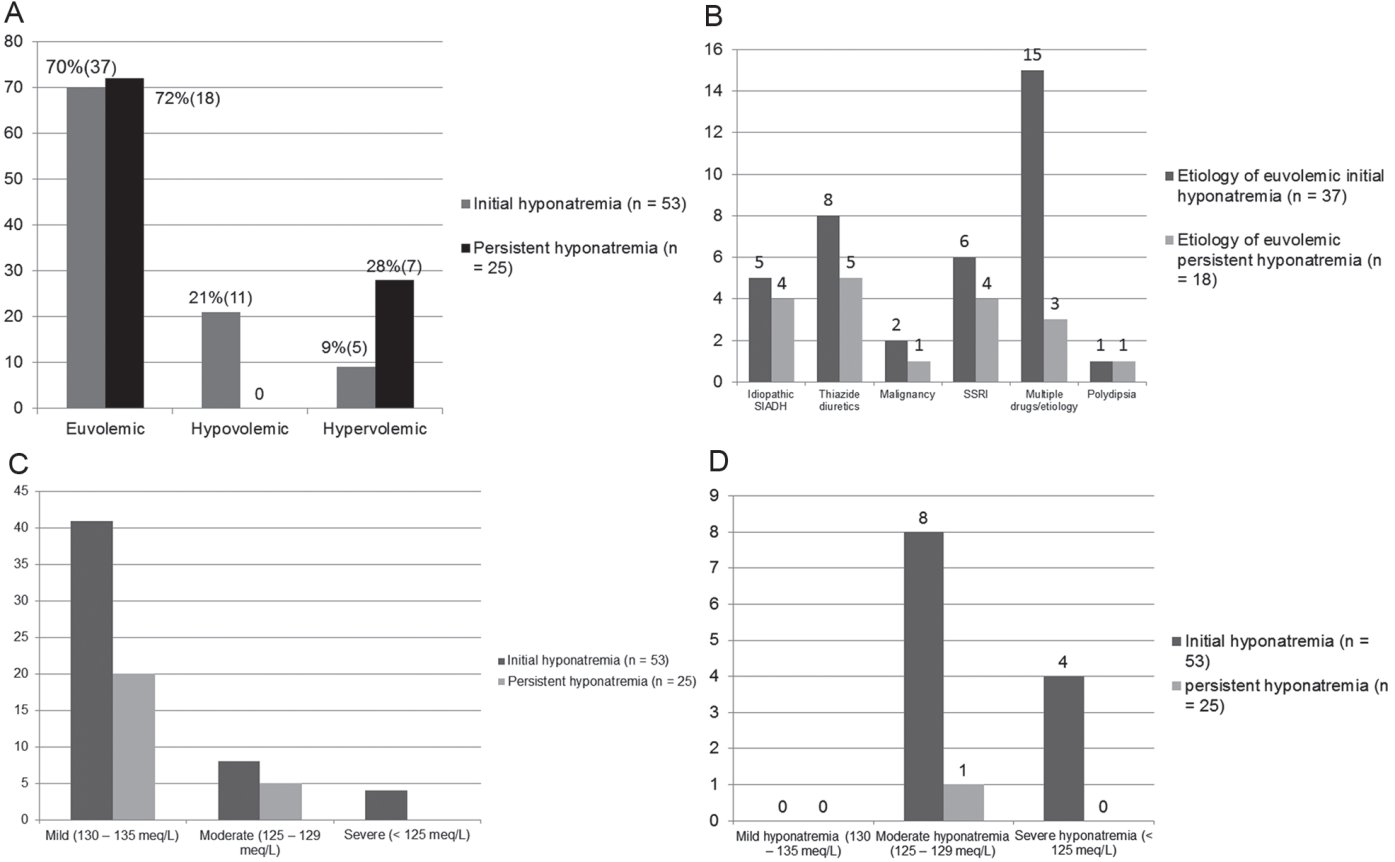
A: Showing the classification of etiology of hyponatremia on the basis of volume. B: Showing the etiology of euvolemic hyponatremia. C: Showing the distribution of severity of hyponatremia. D: Showing the prevalence of symptomatic hyponatremia on the basis of severity.

The etiology of hyponatremia was divided between three categories: euvolemic, hypervolemic, and hypovolemic on the basis of documentation of volume status. Of the 53 patients who had initial hyponatremia, 37 (69.8%) were euvolemic, 5 (9.4%) were hypervolemic, and 11 (20.7%) were hypovolemic (Figure 1A). Of the 25 who had persistent hyponatremia, euvolemic hyponatremia was most common (Figure 1A) and seen in 18 patients (72%) followed by hypervolemia in 7 (28%). There was no documented hypovolemia in those with persistent hyponatremia. Mean sodium value in patients with initial hyponatremia was 131.2 ± 4.5 mEq/L, and in patients with persistent hyponatremia it was 130.4 ± 3.5 mEq/L. The majority of cases of hyponatremia (Figure 1C) were mild and apparently asymptomatic as recognized by the absence of documentation of suggestive neurocognitive dysfunction (Figure 1D). 

In euvolemic initial hyponatremic patients (n = 37), the predominant etiology identified was multiple potential causes (n = 11), thiazides (n = 8) followed by selective serotonin reuptake inhibitor (SSRI) (n = 6), idiopathic SIADH (n = 5), unknown or unclear (n = 4) malignancy (n = 2), and psychogenic polydipsia (n = 1) (Figure 1B). In euvolemic persistent hyponatremic patients (n = 11), the predominant etiology identified was thiazides (n = 5) followed by SSRI (n = 4), idiopathic SIADH (n = 4), multiple potential causes (n = 2), malignancy (n = 1), psychogenic polydipsia (n = 1), and unknown or unclear (n = 1). In patients with initial hyponatremia with multiple potential causes (n = 11), the most common combination was thiazide and SSRI use (n= 4), followed by thiazide and neuroleptic drug use (n = 4), and hypothyroidism with thiazide use (n = 3). In patients with euvolemic persistent hyponatremia with multiple potential causes, a combination of thiazide with neuroleptic use was identified in 2 patients. Initial hypovolemic hyponatremia (n = 11) was associated with symptoms in all patients, and etiologies included diarrhea (n = 2) or diuretics (furosemide n = 6 and thiazide n = 3). In initial hypervolemic hyponatremic (n = 5) end-stage renal disease (ESRD) was seen in 3 and CHF in 2 patients. Amongst persistent hyponatremic patients, no hypovolemia was seen, while the etiology of persistent hypervolemic hyponatremia was CHF in 5 patients (all on diuretics) and ESRD in 2 patients. 

**Figure 2.**
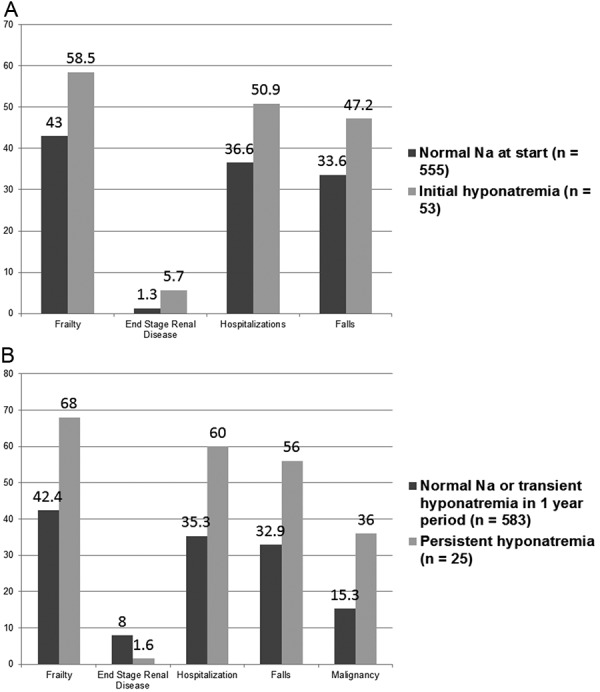
A: Showing demographic and clinical variables significantly associated with initial hyponatremia (percentages). B: Showing demographic and clinical variables significantly associated with persistent hyponatremia (percentages).

When clinical and demographic variables were analyzed on the basis of initial hyponatremia versus normonatremia (Table 2) (Figure 2A), significantly more patients with initial hyponatremia had frailty, ESRD, any hospitalization, and falls. Drugs commonly implicated with hyponatremia, such as diuretics, antipsychotics, or SSRI, were not significantly more present in the initial hyponatremia group. When comparing normonatremic patients or transiently hyponatremic patients over a 1-year period to those patients with persistent hyponatremia (Table 3) (Figure 2B), frailty, length of observation period, malignancy, ESRD, any hospitalization and falls were significantly more in the persistent hyponatremic patients. No difference in mortality was seen in patients when categorized on the basis of initial or persistent hyponatremia. 

**Figure 3.**
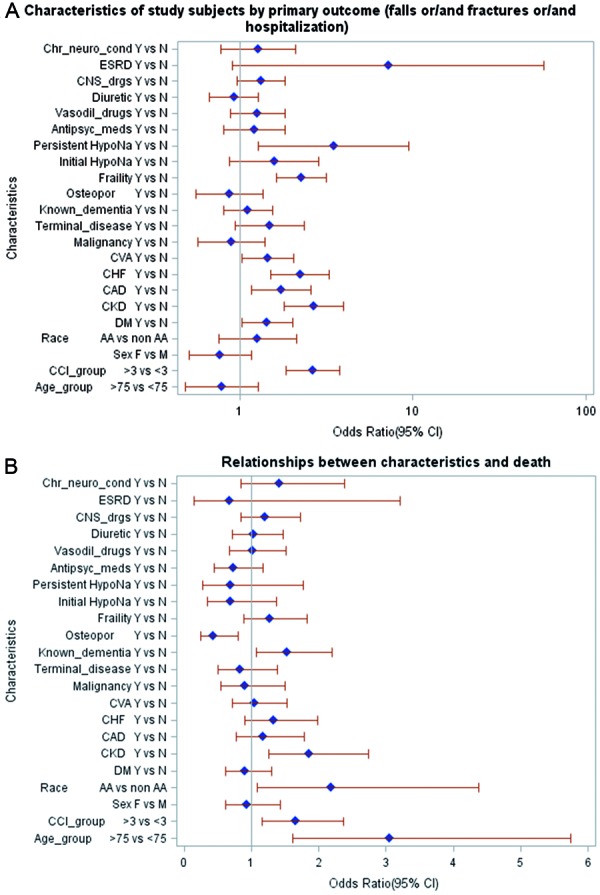
A: Showing subset analysis on factors affecting the composite primary outcome. Baseline hyponatremia refers to initial hyponatremia. B: Showing subset analysis on factors affecting secondary outcome.

The composite primary outcome within 1 year of observation was seen in 56.08% of the total population and on bivariate analysis (Figure 3A); significant factors included Charlson Comorbidity Index (CCI) of > 3, diabetes mellitus, chronic kidney disease, cerebrovascular disease, frailty, persistent hyponatremia (but not initial, transient hyponatremia), and ESRD. However, multivariate logistic regression analysis (Table 4) revealed that CCI, CKD, frailty, and persistent hyponatremia were independent risk factors for the primary outcome. 

Incidence of all-cause mortality was 26.9%. This included patients opting for palliative and hospice care as well as those receiving acute care in the hospital. When a subset analysis was done for overall mortality (Figure 3B), factors that emerged as significant included age of more than 75 years, CCI > 3, African-American race, CKD, frailty, known dementia, and known osteoporosis. Notably, hyponatremia, either initial or persistent, history of malignancy (active or inactive), patient categorized by physician as terminal disease, or frailty were not significantly associated with all-cause mortality. On a multivariate logistic regression analysis (Table 3) advanced age, African-American race, and history of osteoporosis were independently associated with all-cause mortality. 

## Discussion 

Hyponatremia is the most common electrolyte disturbance in the elderly population [1]. Point prevalence in elderly nursing home residents [18] is 18%, while in a 12-month period 53% had 1 or more episodes of hyponatremia. Another inpatient geriatric unit [19] noted a prevalence of 11.3% over a 10-month period, with 4.5% having severe hyponatremia (< 125 mmol/L). In a less ill outpatient population [20], a lower incidence of 7.7% was noted. However, little data exist on the epidemiology of hyponatremia in a frail elderly population. This has important clinical implications as the geriatric population is more prone to this electrolyte disorder due to multiple mechanisms [21, 22, 23, 24, 25]. 

While symptomatic hyponatremia is easily detected and treated, prognosis, therapeutic challenges, and implications of mild chronic hyponatremia have recently been highlighted. Most notable amongst the poor outcomes associated with this condition is the propensity to falls in the setting of neurocognitive dysfunction causing gait ataxia and poor attention span [5]. This has significant implications for geriatric practice given that falls are a common medical problem in the elderly [26], with an annual incidence of 30 – 60% in community-dwelling elders [27]. Consequences of such falls in the elderly [26] include hip fractures, hospitalization, serious head injuries, and admission to long-term care facilities. Furthermore, prospective data from the Rotterdam study [20] showed significant association between baseline mild hyponatremia with recent falls, vertebral, and incidental nonvertebral fractures. Epidemiological and experimental data have shown chronic mild hyponatremia to be an independent risk factor for senile osteoporosis by increasing bone osteoclastic activity in a hyponatremic milieu [8]. Additionally, chronic mild hyponatremia has been associated with increased length of hospital stay, loss of functional independence, and mortality in chronic diseases and in intensive care unit (ICU) settings [9, 20, 28, 29]. 

Our study explored the epidemiology of hyponatremia in a vulnerable elderly home-bound population. The average age of the participants in the population was 84.3 years, which is much higher than previous studies [6, 9, 20, 28]. Variables that we consider clinically relevant in this population and which may have been potential confounders in previous epidemiological associations of hyponatremia were the burden of comorbid illness and the extent of functional limitation due to frailty. Both these parameters were studied using well-validated tools [16, 17]. The incidence of initial hyponatremia was 8.71%, which was slightly higher than 7 7.7% as reported in various studies [20, 30]. The use of a time frame of at least 6 months to define persistent hyponatremia was arbitrary and designed solely to assess the differential effects of initial and persistent hyponatremia on important geriatric outcomes. Ideally, a time-dependent analysis would have given us clear answers as to whether the primary and secondary outcomes were causally related to hyponatremia or not. However, due to the lack of uniformity of biochemical assessment in the study population, we were unable to do so. We chose a short observation period of 1 year as we hypothesized that a short-term and time-dependent association of hyponatremia with geriatric morbidity would strengthen our assumptions of causality. We chose a composite primary outcome as falls, fractures, and hospitalization are often interlinked not just with multiple diseases [31, 32, 33] like CHF, cerebral vascular accident (CVA), and CKD, but also with persistent hyponatremia [33]. 

One of the distinct findings of the study was the variation in the etiology of hyponatremia with respect to duration. While physician-determined euvolemic hyponatremia was the most common cause (69.8% in initial and 72% in persistent hyponatremia), hypovolemic hyponatremia tends to decrease with time (20.75% initial versus 0 in persistent group), while the incidence of hypervolemic hyponatremia increases with time (9.43% in initial versus 28% in persistent hyponatremia). This is partly due to the easy recognition of overt signs and etiology of hypovolemia and its treatment. Not surprisingly, studies in acute settings have reported dehydration (presumably hypovolemia) as the most common cause [34]. We found thiazides to be the most common single identifiable cause for both initial (43.4%) and persistent (28%) hyponatremia, which is consistent with previous studies [6, 20, 34]. Incidence of SSRI-related hyponatremia was ~ 20% regardless of time and agrees well with previous studies [35]. Given the retrospective nature of the study, etiology could not be identified in a large number of patients who were labelled “unknown or unclear”. It is quite possible that many of these patients may have had SIADH. However, this was not clearly investigated. Multiple potential causes were seen in 20.7% of initial and 8% of persistent hyponatremia, which is less than in previous studies in acute settings [6, 34, 36] and in patients with more severe hyponatremia [36]. 

Age-related increase in the incidence of SIADH is a distinct phenomenon that has been previously reported [4, 18, 37]. We found idiopathic SIADH, in 9.4% of patients at baseline and in 16% with persistent hyponatremia, which is similar to 15.2% in previous studies [4]. However, given the nature of the database and the lack of uniformity of renal evaluation for hyponatremia, we believe that SIADH may have been potentially under reported in our study. Acute settings as in institutionalized patients [18] or following falls and fractures [6, 34] have reported a higher incidence of SIADH which could be related to higher exposure to hypotonic fluids unmasking the disease [18] or due to the confounding influence of pain or opioid medications in trauma settings, which are potent stimulus for ADH release [38]. While previous studies had identified advancing age, race (African-American population), smoking, diabetes, CHF, cirrhosis, use of SSRIs or antiepileptics, diuretics of all types, psycholeptics and benzodiazepines, hypoalbuminemia, hypocholestolemia, subclinical hypothyroidism, and even low body mass index as potential risk factors of geriatric hyponatremia [6, 20, 28, 39], the current study identified only frailty and ESRD as significantly associations. This could be due to population heterogeneity across different studies or small sample size of the current study. Of note, hyponatremia in hemodialysis-dependent ESRD has increasingly been reported [40] and is postulated to be due to increased interdialytic free water intake [41]. 

One of the novel findings of the current study is the association of frailty with hyponatremia in the elderly and its emergence as an independent risk factor for primary outcomes in addition to persistent hyponatremia. This is not surprising given the known association of frailty with falls, disability, osteoporosis, admission to hospital, postoperative complications, length of stay in hospital, and even death [16, 42]. Frailty as a clinical syndrome is characterized by progressive sarcopenia or loss of skeletal muscle mass [15]. It is the end result of a number of diseases such as chronic infections, malignancy, and chronic illness causing a systemic inflammatory state with elevated interleukin-6 or C-reactive protein leading to decreased IGF-1 and other hormonal changes leading to weight loss, loss of lean body mass, and endurance. 

There is a paucity of literature on the mechanisms of hyponatremia in frailty. Studies on the hypothalamic/-pituitary adrenal axis in frail elderly patients have shown suboptimal response to ACTH stimulation, suggesting unrecognized adrenal insufficiency as a possible cause of functional limitation and hyponatremia [43]. Another possibility is that the cytokine milieu promoting frailty and functional disability includes interlukin-6 [44], which has been shown, at least in experimental aging animals, to promote increased vasopressin secretion thus impairing fluid homeostasis [45]. Other potential mechanisms could be poor solute intake in frail patients leading to decreased free water excretion by lowering urine osmolarity. Unfortunately, urine osmolarity was not consistently available in the current study to test this hypothesis. On the other hand, it is also plausible that hyponatremia can mediate frailty as sarcopenia has been shown to occur in aging hyponatremic rats [23]. 

Predicting all-cause mortality in an outpatient elderly population is difficult, and a recent systematic-analysis has revealed that most prognostic indices are either biased or weakly generalizable [46]. Contrary to previous observational data, we did not find any association between baseline and persistent hyponatremia with all-cause mortality, although we believe that our study was under powered to test that association robustly [9]. Of note, in a large database study on veteran affairs (VA) patients, Kovedsky et al. [29] demonstrated only a weak association between time-dependent hyponatremia and mortality when analyzed over a 1-year period, which questions the issue of independent causality of mild chronic hyponatremia with mortality. The association between outpatient hyponatremia and increased propensity for hospitalization is also unique in the geriatric population and deserves further studies. 

Some of the strengths of the current study include an attempt to use hyponatremia as a time-dependent and as a baseline variable to investigate its role in contributing to geriatric morbidity. Most studies showing associations have been largely case-control studies [6, 7, 35], while one prospective study analyzed outcomes using initial or baseline hyponatremia. The use of serum sodium in an acute settings like falls and fracture is often confounded by the fact that pain and/or opioid medications in these situations can be potent stimulus for ADH secretion [38]. Our study also identified the varied etiology of chronic hyponatremia with time showing a gradual attrition of hypovolemic hyponatremia and a proportionate increase in the percentage of euvolemic or hypervolemic hyponatremics. This has major etiopathogenic and therapeutic implications. We were also able to identify newly described risk factors for hyponatremia, including frailty and end-stage renal disease. Given the profound implications of frailty in geriatric morbidity and mortality, we believe that this may be a potential confounding variable in many studies showing associations with baseline hyponatremia. 

Some weaknesses of the current study include a small sample size, short follow-up period, and the fact that data were derived from a single center. Also, the retrospective nature of the data has inherent risks of being subject to selection and information bias. We tried to limit information bias with a detailed protocol for data collection and simultaneous data collection by two independent workers to ensure reproducibility of data. The data on etiological assessment of hyponatremia are also limited by the lack of a uniform protocol for renal consults or laboratory assessment. This is especially significant as volume assessment in hyponatremia by physical examination has poor sensitivity and specificity [47]. As a consequence of the small sample size, the impact of different etiologies of hyponatremia could not be analyzed. It is also not clear as to how many of the hyponatremic patients were truly asymptomatic. Furthermore, detailed neurological assessment in patients who experienced a fall was not available to ascertain the presence of subtle neurocognitive defects from hyponatremia. Finally, the study was under powered to assess the association of hyponatremia with all-cause mortality. We hope that the limitations of the current study will serve as a fresh ground for newer research in geriatric hyponatremia. 

Despite all its shortcomings, we feel that our study is significant in shedding light on the epidemiology of hyponatremia in a very elderly population where the burden of disabling comorbid conditions is substantial yet patients are not at risk of imminent death. Given the significant association between frailty and hyponatremia, we believe we have identified a potential confounder in epidemiological studies on hyponatremia which may also serve as the elusive etiologic link between hyponatremia and its poor prognostic implications in the geriatric population. The current study also underscores the need to use serum sodium as a time-dependent variable to further investigate the issue of causality. 

## Acknowledgment 

The authors wish to thank the biostatistical team from the Medstar Research Institute, Washington, DC for their help in data analysis. The authors also thank the Office of the Graduate Medical Education, Georgetown University/Washington Hospital Center for funding the bio statistical analysis of the study. Furthermore, the authors would like to state that they have no financial interests, affiliation, or relationship attached to the conduct, analysis, or publication of the current research work. 

**Table 1.** Demographic and clinical variables in the study population (n = 608).

Characteristics	Values (percentage)
Age (years)	84.3 ± 9.3
Observation period (months)	41.5 ± 20.5
CCI	2.8 ± 2.2
Female	469 (77.1%)
Race	
African American (AA)	543 (89.5%)
Caucasian	45 (7.4%)
Hispanic	6 (0.99%)
Asian	4 (0.66%)
Unknown	9 (1.5%)
Frailty	268 (44.4%)
DM	215 (35.4%)
HTN	527 (86.7%)
CKD	162 (26.6%)
CAD	139 (22.9%)
CHF	160 (26.3%)
CLD	5 (0.8%)
COPD	95 (15.6%)
CVA	196 (32.2%)
Hypothyroidism	84 (13.8%)
Terminal disease	94 (15.6%)
Malignancy	91 (14.9%)
Psychiatric disease	197 (32.5%)
Dementia	266 (44.1%)
Chronic neurological conditions	75 (12.3%)
Osteoporosis	92 (15.2%)

Mean ± SE. CCI = Charlson comorbidity index; DM = diabetes mellitus; HTN = hypertension; CKD = chronic kidney disease; CAD = coronary artery disease; CHF = congestive heart failure; CLD = chronic liver disease; COPD = chronic obstructive pulmonary disease; CVA = cerebrovascular disease.

**Table 2.** Clinical variables in the study populations analyzed on the basis of baseline hyponatremia.

Characteristics	Normal at baseline (n = 555)	Initial hyponatremia (n = 53)	p-value
Age	84.1 ± 9.26	86.3 ± 9.7	0.0694
Observation period	42.0 ± 20.4	36.9 ± 20.1	0.0769
CCI	2.7 ± 2.1	3.0 ± 2.6	0.8688
Female	427 (76.9%)	42 (79.3%)	0.7022
Race			0.8765
AA	496 (89.4%)	47 (88.7%)	
Non-AA	59 (10.6%)	6 (11.3%)	
Frail	237 (43.0%)	31 (58.5%)	0.0303*
DM	199 (36.8%)	16 (30.2%)	0.4097
HTN	480 (86.5%)	47 (88.7%)	0.6536
CKD	151 (27.2%)	11 (20.8%)	0.3100
CAD	130 (23.4%)	9 (17.0%)	0.2860
CHF	147 (26.5%)	13 (24.5%)	0.7571
Vasodilator drugs	403 (72.6%)	37 (69.8%)	0.6630
Osteoporosis	84 (15.2%)	8 (15.1%)	0.9895
CVA	178 (32.1%)	18 (34.0%)	0.7785
Hypothyroidism	77 (13.9%)	7 (13.2%)	0.8892
Terminal disease	86 (15.6%)	9 (17.0)	0.7931
Malignancy	79 (14.2%)	12 (22.6%)	0.1012
Psychiatric disease	177 (32.0%)	20 (37.7%)	0.3900
Diuretic	269 (48.6%)	28 (52.8%)	0.5521
Antipsychotic meds	108 (19.5%)	9 (17.3%)	0.7068
SSRI	131 (23.6%)	15 (28.3%)	0.4443
CNS drugs	296 (53.3%)	28 (53.9%)	0.9435
ESRD	7 (1.3%)	3 (5.7%)	0.0484*
Known dementia	246 (44.7%)	20 (37.7%)	0.3276
Chronic neuro condition	68 (12.3%)	7 (13.2%)	0.8399
Fractures	29 (6.9%)	4 (9.3%)	0.5513
Hospitalization	203 (36.6%)	27 (50.9%)	0.0393*
Falls	186 (33.6%)	25 (47.2%)	0.0471*
Mortality	153 (27.6%)	11 (20.8%)	0.2857

Mean ± SE, *statistically significant (p < 0.05). CCI = Charlson comorbidity index; DM = diabetes mellitus; HTN = hypertension; CKD = chronic kidney disease; CAD = coronary artery disease; CHF = congestive heart failure, CNS = central nervous system; CVA = cerebrovascular disease; SSRI = selective serotonin reuptake inhibitor; ESRD = end-stage renal disease.

**Table 3.** Clinical variables in the study populations analyzed on the basis of presence or absence of persistent hyponatremia.

Characteristics	Normal Na over 1-year follow up (n = 583)	Persistent hyponatremia (n = 25)	p-value
Age	84.6 ± 9.2	84.7 ± 10.3	0.9729
Observation period	41.1 ± 20.1	31.3 ± 16.8	0.0236*
CCI	2.8 ± 2.1	3.3 ± 2.7	0.4217
Female	386 (76.4%)	20 (80.0%)	0.6811
Race			0.7508
AA	449 (88.9%)	22 (88.0%)	
Non AA	56 (11.1%)	3 (12.0%)	
Frail	213 (42.4%)	17 (68.0%)	0.0116*
DM	186 (36.8%)	7 (28.0%)	0.3704
HTN	441 (87.3%)	21 (84.0%)	0.5472
CKD	147 (29.1%)	4 (16.0%)	0.1800
CAD	123 (24.4%)	5 (20.0%)	0.6193
CHF	135 (26.7%)	5 (20.0%)	0.4561
Vasodilator drugs	374 (74.1%)	18 (72.0%)	0.8188
Osteoporosis	77 (15.3%)	4 (16.0%)	1.0000
CVA	166 (32.9%)	9 (36.0%)	0.7454
Hypothyroidism	76 (15.1%)	2 (8.0%)	0.5604
Terminal disease	66 (13.2%)	6 (24.0)	0.1243
Malignancy	77 (15.3%)	9 (36.0%)	0.0060*
Psychiatric disease	156 (31.0%)	11 (44.0%)	0.1707
Diuretic	257 (51.0%)	10 (40.0%)	0.2833
Antipsychotic meds	96 (19.0%)	6 (25.0%)	0.4674
SSRI	118 (23.4%)	7 (28.0%)	0.5942
CNS drugs	271 (53.7%)	15 (62.5%)	0.3960
ESRD	8 (1.6%)	2 (8.0%)	0.0214*
Known dementia	224 (44.6%)	11 (44.0%)	0.9513
Chronic neuro condition	60 (11.9%)	4 (16.0%)	0.5271
Fractures	26 (6.2%)	3 (15.8%)	0.1246
Hospitalization	178 (35.3%)	15 (60.0%)	0.0121*
Falls	166 (32.9%)	14 (56.0%)	0.0171*
Mortality	158 (31.3%)	6 (24.0%)	0.6176

Mean ± SE, *statistically significant (p < 0.05). CCI = Charlson comorbidity index; DM = diabetes mellitus; HTN = hypertension; CKD = chronic kidney disease; CAD = coronary artery disease; CHF = congestive heart failure; CNS = central nervous system; CVA = cerebrovascular disease; SSRI = selective serotonin reuptake inhibitor; ESRD = end-stage renal disease.

**Table 4.** Multivariate logistic regression analysis.

Multivariate logistic regression analysis of the primary outcome after adjusting for all the identified potential confounders found in subset analysis							Multivariate logistic regression analysis of the secondary outcome (all-cause mortality) after adjusting for all the identified potential confounders in subset analysis						
Analysis of effects				Odds ratio estimates			Analysis of effects				Odds ratio estimates		
Effect	DF	Wald χ^2^	Pr > χ^2^	Point estimate	95% Wald Confidence Limits		Effect	DF	Wald χ^2^	Pr > χ^2^	Point estimate	95% Wald confidence limits	
CCI	1	1.1230	0.2893	1.313	0.793	1.953	Age	1	9.9050	0.0016*	2.883	1.491	5.574
DM	1	0.5735	0.4489	1.178	0.771	1.585	CCI	1	0.4077	0.5231	1.161	0.734	1.839
CKD	1	6.3817	0.0115*	1.934	1.159	2.709	Race	1	3.9243	0.0476*	2.086	1.008	4.316
CAD	1	1.5093	0.2192	1.356	0.834	1.878	CKD	1	2.6618	0.1028	1.504	0.921	2.454
CHF	1	6.8798	0.0087*	1.891	1.175	2.607	Known dementia	1	3.3409	0.0676	1.434	0.974	2.111
CVA	1	1.3361	0.2477	1.290	0.838	1.742	Osteoporosis	1	7.1156	0.0076*	0.425	0.226	0.797
Frail	1	18.0019	< 0.0001*	2.327	1.575	3.424							
Persistent hyponatremia	1	5.4986	0.0190*	3.524	1.230	5.818							
ESRD	1	0.3617	0.5475	1.944	0.223	3.665							

*p < 0.05, significant. CCI = Charlson comorbidity index; DM = diabetes mellitus; DF = degrees of freedom; CHF = congestive heart failure; CKD = chronic kidney disease; CAD = coronary artery disease; CVA = cerebrovascular disease; ESRD = end-stage renal disease.
